# Parkinson’s disease: Impact of clinical and cognitive aspects on quality of life

**DOI:** 10.1590/S1980-57642010DN40200010

**Published:** 2010

**Authors:** Glória Maria Almeida Souza Tedrus, Lineu Correa Fonseca, Patrícia Mencaroni Kange

**Affiliations:** 1 Professor of Neurology, Pontifícia Universidade Católica de Campinas, Campinas SP, Brazil (PUC-Campinas), Scholarship holders.; 2 Student of Faculty of Medicine with FAPIC placement scholarship - Catholic University of Campinas (PUC-Campinas), Campinas SP, Brazil.

**Keywords:** Parkinson’s disease, quality of life, depression, cognition

## Abstract

Parkinson’s disease (PD) is a chronic disease manifested principally by motor signs and symptoms, but with frequent neuropsychological alterations.

**Objectives:**

To study the relationship between clinical and cognitive aspects and the perception of quality of life (QOL) in PD patients.

**Methods:**

Twenty consecutive patients (13 men) with idiopathic PD (mean age: 64.5y), mean disease time of 7.8 years and at stages 1-3 according to the modified Hoehn and Yahr staging scale (HYS), all outpatients from the Neurology Department of the Celso Pierro General and Maternity Hospital (PUC-Campinas), were analyzed. The following were applied: a clinical-neurological assessment, the Mini-Mental State Examination (MMSE), standard neuropsychological battery of the CERAD (Consortium to Establish a Registry for Alzheimer’s Disease), Hamilton Depression Rating Scale (HAM-D) and a QOL questionnaire (Parkinson’s Disease Questionnaire - PDQ-39). Statistical analysis was carried out at a significance level of p<0.05.

**Results:**

On the PDQ-39 under the sections total, mobility and activities of daily living, and the items motor compromise (HYS) and language of the MMSE were predictors of worse QOL. Verbal fluency was a factor for emotional well-being on the PDQ-39, whereas higher scores for HAM-D and worse performance on the item attention and calculation of the MMSE were associated with worse QOL in the social support section. Total score on the MMSE and educational level were QOL factors in cognition.

**Conclusions:**

The findings of the present study suggest that clinical, cognitive, motor or other depression-related factors contribute differently to the domains of QOL.

## Abstract (pt)

Doença de Parkinson (DP) é uma doença crônica que se manifesta principalmente por sinais e sintomas motores, mas com alterações neuropsicológicas frequentes.

**Objetivos:**

Estudar a relação de aspectos clínicos e cognitivos com a percepção da qualidade de vida (QV) em pacientes com DP.

**Métodos:**

Foram analisados 20 pacientes (13 do gênero masculino) consecutivos com DP idiopática (idade média: 64,5 anos), tempo médio de doença de 7,8 anos e estágio 1-3 na escala Hoehn e Yarh modificada (HY), procedentes do ambulatório de Neurologia do Hospital e Maternidade Celso Pierro (PUC-Campinas). Foram realizados: anamnese, exame neurológico, mini-exame do estado mental (MEEM), bateria neurospicológica do CERAD, escala de depressão de Hamilton (EDH) e questionário de QV (Parkinson’s Disease Questionnaire - PDQ-39). Foi feito o estudo estatístico, com nível de significância p<0.05.

**Resultados:**

Comprometimento motor e no item “linguagem” do MEEM foram preditores de pior QV no PDQ-39 “total”, “mobilidade” e “atividades da vida diária”. A fluência verbal foi fator de “bem estar emocional” do PDQ-39, enquanto maiores escores na EDH e desempenho inferior no item “atenção e calculo” do MEEM estiveram associados a pior QV em “apoio social”. O escore total do MEEM e o nivel de escolaridade foram fatores de QV em “cognição”.

**Conclusões:**

Nossos achados sugerem que diferentes fatores clínicos, cognitivos, da motricidade ou ligados a depressão contribuem de modo diferenciado para os diversos domínios da QV.

Parkinson’s disease (PD) is a neurodegenerative condition causing functional degeneration and compromised psychosocial status. The motor aspects of PD are well known, however the physiopathology of the non-motor neuro-psychiatric manifestations leading to physical, mental and socioeconomic repercussions and considerable negative impact on the quality of life (QOL) of both patients and caregivers have also been discussed.^1-6^

Studies on QOL in PD have been carried out in various countries,^7-9^ and more recently in Brazil.^10-13^

Of the instruments employed to evaluate quality of life, the most frequently used is the Parkinson’s Disease Questionnaire (PDQ-39), which is specific, easy to apply and reproduce,^8^ and has been validated for the Brazilian population.^10^

Some studies have indicated that compromise of QOL stems from various factors, the most important of which is degree of motor compromise,^2-4,14,15^ whereas other authors have indicated depression as a major factor.^2-4,10,15-18^ The findings concerning the influence of gender,^4,10,16^ age^2-4,16^ and educational level,^10,15^ amongst others, are not unanimous.^3,14,17,19^

It is known that cognitive aspects can aggravate the evolution of PD, increase treatment costs and overburden caregivers.^16^ However, there are differences between studies on QOL evaluations in overall PD series, exclusively in non-demented patients^4,19,20^ and using different neuropsychological procedures.^2,6,16^

Identification of those factors and disabilities having the greatest impact on QOL in patients with PD could help in determining the best intervention strategy.^14,18^

The majority of the studies to date have considered factors related to the total PDQ-39 score, and only a few have attempted to evaluate the diverse factors of QOL in connection with the different domains of the PDQ-39.^12,13^

The objective of the present study was to evaluate the impact of clinical aspects, results from the Hamilton Depression Rating Scale, as well as cognitive aspects, on the perception of QOL in an integrated manner for the various domains of the PDQ-39.

## Methods

### Subjects

Twenty consecutive patients (13 men) with a mean age of 64.5 years old (SD-10.4y) and a probable or definitive diagnosis of PD according to the Calne et al.^21^ criteria, all outpatients at the Neurology Clinic of the Celso Pierro General & Maternity Hospital (PUC-Campinas), were included. The patients were taking dopamine and were evaluated in the on phase.

The study was approved by the Ethics Committee for Research in Humans of PUC-Campinas under protocol 407/08, and all the patients signed a term of consent.

### Procedures

The procedures were: a clinical-neurological assessment; modified Hoehn-Yahr scale (HYS); standard neuropsychological battery of the Consortium to Establish a Registry for Alzheimer’s Disease (CERAD), Hamilton Depression Rating Scale (HAM-D) and the Parkinson’s Disease Questionnaire 39 (PDQ-39).

Motor disability was graded using the modified Hoehn and Yahr staging scale (HYS).^22,23^

The standard neuropsychological battery of CERAD, used in the cognitive assessment, comprised a semantic verbal fluency assessment, abridged Boston Naming Test (15 items), the Mini-Mental State Examination (MMSE), word list memory with repetition, recall and recognition, and constructional praxis with copy and recall.^24^ The applicability of the battery to the Brazilian population had been previously certified.^25^

The Hamilton Depression Rating Scale (HAM-D)^26^ is a depression scale used to quantify the presence and severity of the symptoms which adopts objective, pre-established criteria and supplements the clinical evaluation. This is the most commonly used observer scale in clinical practice and has been indicated to evaluate depression in PD. Although the author of the scale did not indicate cutoff points, in practice, scores above 25 points indicate severe depression, 18-24 moderate depression, 7-17 mild depression and below 6 as the absence of depression.^27^

The Parkinson’s Disease Questionnaire 39 - PDQ-39^8^ is used to evaluate QOL in individuals with PD, is easy to apply and reproduce and has been used to evaluate the efficacy of treatment and impact of the disease on the QOL of PD patients. The questionnaire was adapted for Brazilian Portuguese at the Health Services Research Unit - Oxford in 2005 and validated for the Brazilian population in 2007.10 The PDQ-39 consists of 8 domains (scales): mobility, activities of daily living, emotional well being, stigma, social support, cognitions, communication and bodily discomfort. The total score ranges from 0 to 100 and the lower the score the greater the perception of QOL.^8^ The PDQ-39 was applied in the form of an interview (carried out by the author: PMK) so as to avoid errors of interpretation, since the majority of the participants had a low level of education. The mean application time was 15 minutes.

Patients suffering from cognitive deficit that compromised their understanding of the questions or that presented other chronic incapacitating diseases were excluded.

### Data analysis

Kendall s tau coefficient was used to analyze the correlation between the socio-demographic, clinical and cognitive aspects and perception of QOL indicated by the total score on the PDQ-39 and the 8 domains.

Multiple regression analyses were carried out to determine which factors contributed most to the PDQ-39 scores (dependent variables), using the variables yielding p<0.10on the respective prior correlation analyses (independent variables).

Stepwise linear regression was performed to identify the statistically significant predictors of the PDQ-39 domains, using the Statistical Package for the Social Sciences (SPSS), version 10. A level of significance of p<0.05was used in the statistical analyses.

## Results

### Clinical e cognitive aspects and perception of QOL

Table 1 shows the socio-demographic and clinical data along with the results obtained for the CERAD, HAM-D and PDQ-39.

**Table 1:** Socio-demographic, clinical and cognitive aspects, Hamilton depression scale, and PDQ-39 scales in 20 patients with PD.

Variable	Mean	SD	Min	Max
Age (years)	64.5	10.4	47	86
Educational level (years)	4.2	4.1	0	17
Duration of PD (years)	7.8	5.5	1	25
Hoen & Yahr scale	1.85	0.7	1.0	3.0
Verbal fluency	12.3	4.6	2	21
Abridged Boston naming test	11.4	3.6	0	15
Word list memory with repetition	12.5	5.5	0	21
Word list recall	4.2	2.7	0	8
Constructional praxis	1.4	1.3	0	5
Word list recognition	6.6	3.5	0	10
Constructional praxis recall	2.0	1.5	0	5
Mini-Mental State Examination	23.2	6.6	9	30
Attention and calculation	2.3	2.1	0	5
Language	6.7	2.2	1	8
Time orientation	4.2	1.7	0	5
HAM-D	14.7	7.2	4	29
PDQ-39 scales				
Mobility	39.7	34.5	0	100
Activities of daily living	38.7	32.7	0	100
Emotional well being	36.7	23.7	0	83
Stigma	27.5	35.1	0	100
Social support	33.7	26.5	0	75
Cognitions	32.8	25.8	6.3	100
Communication	35.4	29.3	0	83.3
Bodily discomfort	47.1	26.3	0	100
Total	36.9	20.9	9.6	7.4

HAM-D, Hamilton Rating Scale for Depression.

According to the results of the HAM-D, 17(85%) of the PD patients were depressed, of which 12(60%) had mild symptoms, 2 moderate symptoms and 3 severe depression.

According to the PDQ-39 there was perception of worse QOL for the domains of mobility, activities of daily living or bodily discomfort.

### Relation between PDQ-39 and socio-demographic and clinical aspects

There were no significant differences in the PDQ-39 with respect to gender (Mann-Whitney U test).

Table 2 shows the correlation coefficients (Kendall’s rank correlation-tau-b) for the relation between the PDQ-39 domains, socio-demographic and clinical data and results of the HAM-D.

**Table 2:** Correlation coefficients for PDQ-39 (total and domains) and age, educational level, duration of PD, Hoen & Yahr scale, Hamilton Rating Scale for Depression, for the 20 patients with PD.

	Correlation coefficient (Kendall's tau-b)				
	Age	Educational level	Duration of PD	Hoen & Yahr scale	HAM-D
Mobility	0.381*	-0.124	0.160	0.465**	0.202
Activities of daily living	0.139	0.041	0.027	0.482**	0.174
Emotional well being	0.048	-0.047	0.182	0.205	0.104
Stigma	-0.148	0.216	0.104	0.107	0.078
Social support	0.133	0.000	0.034	0.213	0,441*
Cognitions	0.023	0.145	0.012	0.270	0.243
Communication	0.089	0.129	0.012	0.282	0.000
Bodily discomfort	0.256	-0.012	0.103	-0.111	-0.033
Total	0.222	-0.047	-0.118	0.406*	0.247

Kendall's rank correlation (tau-b);

* p<0.05;

** p<0.01; HAM-D, Hamilton Rating Scale for Depression.

There was a statistically significant perception of greater QOL compromise with respect to “total”, “mobility” and “activities of daily living” scores for patients with higher scores on the HYS scale i.e. those with more compromised mobility. In addition, greater QOL compromise with respect to social support was found for those with more complaints of depression on the HAM-D (Table 2).

No significant correlation was observed between PDQ-39 score and age, educational level or duration of PD (Table 2). There was also no correlation between QOL and the dose of L-dopa taken.

### Relation between PDQ-39 and cognitive aspects

Table 3 shows the results of the Kendall’s rank correlation-tau-b analysis for PDQ-39 and cognitive aspects.

**Table 3:** Values for correlations between scores on the PDQ-39 domains and scores on cognitive aspects (CERAD).

	MMSE						CERAD others			
	Total	Time orientation	Space orientation	Attention calculation	Language		Verbal fluency	Word list recall	Constructional praxis	Word list recognition
Mobility	-0.379*	-0.327	-0.278	-0.392*	-0.394*		-0.261	-0.267	0.263	-0.267
Activities of daily living	-0.301	-0.327	-0.073	-0.314	-0.320		-0.136	-0.124	0.206	-0.124
Emotional well being	-0.181	-0.416*	-0.278	-0.321	-0.221		-0.467**	-0.187	0.232	-0.187
Stigma	0.018	0.069	0.380	-0.007	0.022		0.138	0.132	0.048	0.132
Social support	-0.336	-0.266	-0.093	-0.475**	-0.409*		-0.434*	-0.118	0.286	-0.118
Cognitions	-0.280	-0.216	-0.147	-0.505**	-0.284		-0.398*	-0.235	0.186	-0.235
Communication	-0.165	-0.110	0.008	-0.111	-0.236		-0.124	-0.094	0.156	-0.094
Bodily discomfort	0.123	0.378*	0.100	0.097	0.048		0.221	0.110	-0.019	0.110
Total	-0.374*	-0.288	-0.185	-0.452**	-0.416*		-0.349*	-0.201	0.358*	-0.201

Kendall's rank correlation (tau-b);

* p<0.05;

** p<0.01; MMSE, Mini-Mental State Examination.

There was a statistically significant correlation between the perception of greater QOL compromise in total PDQ-39 and cognitive compromise as evaluated by the total score for performance on the MMSE and also for the sub-items “attention and calculation”, “language”, “verbal fluency” and “constructive praxis” (Table 3).

A significant correlation was observed between greater compromise in the mobility domain and poor performance on the total score for the MMSE and on the sub-items “attention and calculation” and “language” (Table 3).

The perception of worse quality of life in “emotional well being” was shown to significantly correlate with poor performance in “time orientation” and “verbal fluency”, whereas worse quality of life in “social support” and “cognitions” was associated with poor performance in “attention and calculation” and “verbal fluency”. Poor performance on the “language” item of the MMSE was also associated with worse quality of life in “social support” (Table 3).

### PDQ-39 predictors; multiple regression analysis

Table 4 shows the variables maintained as significant predictors for total PDQ-39 and its domains on the multiple regression analyses, and also the adjusted values for the coefficient R^2^, the standardized coefficient, 95% CI and p value.

**Table 4:** Multiple regression for the PDQ-39 scale scores: predictor variables with significant effects for the 20 patients with PD.

Dependent variable PDQ-39 scale scores	Significant predictor	Adjusted R^2^	Coefficient	Standardized coefficient
Mobility	HYSLanguage	0.507	19.2-7.8	0.415-0.499
Activities of daily living	HYSLanguage	0.460	19.4-6.5	0.441-0.440
Emotional well being	Verbal Fluency	0.340	-3.1	-
Stigma	Not significant	-	-	-
Social support	HAM-DAttention and calculation	0.402	1.4-5.5	0.403-0.431
Cognitions	MMSEEducational level	0.558	-3.53.1	-0.8880.484
Communication	Not significant	-	-	-
Bodily discomfort	Not significant	-	-	-
Total	HYSLanguage	0.562	10.8-5.0	0.385-0.527

HYS, modified Hoehn and Yahr staging scale; MMSE, Mini-Mental S tate Examination; HAM-D, Hamilton Rating Scale for Depression

The Hoehn and Yahr staging scale and the score for “language” on the MMSE were predictors for the total score on the PDQ-39 and also for the sub-items of “mobility” and “activities of daily living”.

Verbal fluency was significant on the model for “emotional well being”, whilst HAM-D and “attention and calculation” were significant for “social support”. Total MMSE score and “educational level” were predictors for the “cognitions” domain of the PDQ-39.

## Discussion

Recently there has been growing interest in evaluating QOL in PD patients.^7,14^ QOL scales, as are those of the PDQ-39,tend to be subjectively based and reflect information provided by the patient with respect to their health, including emotional and social aspects. These can therefore serve to contribute to medical orientation^17^ if evaluated with the fitting criteria.^2^

PD patients are known to present worse perceived QOL on physical, social and emotional dimensions compared with healthy members of the population or with those with other chronic diseases such as heart disease.^3,4,18^

In the present study there was perception of worse QOL in the dimensions “mobility”, “activities of daily living” and “bodily discomfort” of the PDQ-39. These results are in agreement with the findings of other studies^3,4,8,11-13,15,18,28^ which have associated the fall in QOL with dimensions related to physical aspects.

The limited number of patients involved in the present study represents a limitation, particularly in the correlation and multiple regression analyses.

### Relationship between socio-demographic data and perception of QOL

There was a positive correlation between the perception of worse QOL and age in the present study, although in the multiple regression analysis age did not retain a significant effect. Some studies have shown worsening QOL with age,^2^ although results from studies regarding the influence of age^2-4,16,29^ and gender^3,4,16,29^ are conflicting.

The influence of educational level on QOL has been highlighted by several authors^10^ but is not unanimous.^15^ In the present study, educational level only was a predictor only of the “cognitions” domain, whereas in research by Carod-Artel et al.^10^ the relation between educational level and QOL was observed in various domains of the PDQ-39.

Advanced age at disease onset and time of the disease, both isolated and in association, have been associated to worse QOL on the PDQ-39 (total or some domains)^2,4,10^ although this relationship is not always observed.^10,13,15,18^

The impact of PD on QOL has been shown to increase in the more advanced stages of the disease in longitudinal studies, but the relationship with time of disease is weak^4,18^.

### Relationship between clinical aspects and QOL perception

The present study showed perception of greater QOL compromise (total PDQ-39 and in the dimensions of “mobility” and “activities of daily living”) in those patients with more severe motor involvement, similar to the findings of other studies.^2-5,10,14,15,28,29^

The motor involvement characterized by the HYS was a predictor for the items “mobility” and “activities of daily living” and an important factor in the PDQ-39. Stronger correlations between HYS and the PDQ-39 domains have been reported in the literature.^12^

### Relationship between depression and QOL perception

Depression has been indicated as the factor that most contributes to worse QOL.^2,4,13-16,28,30^ However, some studies found no relationship between depression and compromised QOL,^29,31^ whereas in others the present study included, this relationship was restricted to only some of the domains.^18^

These discrepancies in the relationship between depression and QOL across different studies could be explained in part by the use of distinct instruments that consider or otherwise, hallmarks of PD such as hypomimia, motor slowing and attention disorders, as being manifestations of depression.^15,16^ In a study carried out in 787 PD patients, only 1% of the cases showed clinical complaints of depression although 50% had been considered depressed after applying a depression scale, confirming the different aspects of characterizing depression.^14^

In the present study, statistically significant positive correlation was found only between the domain “social support” of the PDQ-39 and HAM-D, whereas other authors^13^ described relationships with various domains.

### Relationship between cognitive aspects and QOL perception

In the present study, cognitive aspects were evaluated that included the elements recommended by the publication “Diagnostic procedures for Parkinson’s disease dementia: recommendations from the Movement Disorder Society Task Force” which are, respectively: for attention, the serial 7s of the MMSE; executive function, verbal fluency; visuo-constructive ability, MMSE pentagons, and for memory, word recall of the MMSE.^32^

Although some publications failed to confirm a relationship between cognitive compromise and worsened QOL,^15,18^ this link was found in the present study, in agreement with the majority of other studies.^2-4,16,20^

There is consensus in the literature concerning the substantial negative impact of cognitive compromise, especially that of dementia, on QOL and mortality rate among PD patients.^4,33^ On the other hand, although an association between the severity of motor compromise and occurrence of cognitive disorders is recognized,^34,35^ there is no consensus on the subject.^36^

In the present study, significant correlation was observed between worse perception of total QOL and total MMSE score, “verbal fluency”, “attention and calculation”, “language” and “constructional praxis”.

A range of cognitive abilities can be compromised in PD,^34^ and verbal fluency is recognized as a risk factor for the development of dementia in this patient group.^37^ In the present study, akin to that of Muslimovic et al.,^38^ verbal fluency was found to be significantly related to greater compromise of QOL in total PDQ-39 and the social support dimension.

It is important to highlight that greater alterations in “attention and calculation” and “constructional praxis” comprise the differentiated profile of PD with dementia versus that of Alzheimer’s disease,^39^ and it was these aspects that showed greatest association with QOL.

The item “language” of the MMSE showed a strong association with QOL in the present study, entering the multiple regression models with respect to total PDQ-39, “mobility” and “activities of daily living”. This may have occurred because it reflected cognitive compromise occurring later in the disease course and was thus associated with greater severity of the disease.

Relationships between specific aspects of cognition and the different domains of the PDQ-39 are rarely analyzed in the literature.

In conclusion, the findings of the present study suggest that different clinical, cognitive or motor factors, or those connected to depression, contribute in differentiated ways to the various domains of QOL.

Prospective and longitudinal studies are needed to better evaluate QOL in PD patients in an effort to understand the factors that influence the progression of the disease.
